# Pre-exposure Prophylaxis (PrEP) Uptake Among Older Individuals in Rural Western Kenya

**DOI:** 10.1097/QAI.0000000000002150

**Published:** 2019-08-30

**Authors:** Winter A. Olilo, Maya L. Petersen, Catherine A. Koss, Eric Wafula, Dalsone Kwarisiima, Kevin Kadede, Tamara D. Clark, Craig R. Cohen, Elizabeth A. Bukusi, Moses R. Kamya, Edwin D. Charlebois, Diane V. Havlir, James Ayieko, SEARCH Collaboration

**Affiliations:** a Centre for Microbiology Research, Kenya Medical Research Institute, Nairobi, Kenya; b University of California, Berkeley, Berkeley, CA; c University of California, San Francisco, San Francisco, CA; d Makerere University, Kampala, Uganda

***To the Editors:***

## INTRODUCTION

HIV pre-exposure prophylaxis (PrEP) markedly reduces risk of HIV acquisition and is thus an important tool for HIV prevention.^1,2^ PrEP implementation has largely focused on perceived high-risk groups such as men who have sex with men,^3^ sex workers,^4,5^ and adolescent girls and young women^6,7^ in high HIV prevalence settings. However, each year, 100,000 adults aged 50 years and older are newly infected with HIV, of whom 74% live in sub-Saharan Africa.^8^ Older adults may be particularly vulnerable to HIV acquisition due to postmenopausal hormonal changes among women and intergenerational sex.^9,10^ In addition, in some regions of Kenya, practices such as widow inheritance (cultural “cleansing” after the death of a husband that involves sexual practices which may be unsafe)^11,12^ may increase risk of HIV acquisition across men and women of all ages. However, older adults have not been a major focus of HIV prevention efforts, and little is known about their sexual risk behavior, HIV risk perception, and willingness to initiate PrEP. Within the population-based Sustainable East African Research in Community Health (SEARCH) trial, we sought to evaluate HIV risk perception, uptake of PrEP, and factors associated with PrEP uptake among individuals aged 45 years and older in rural Kenya.

## METHODS

### Study Setting and Population

Between July 2016 and May 2017, the SEARCH Study (NCT01864603) conducted population-based HIV testing, assessed HIV acquisition risk, and offered PrEP in 6 rural communities of approximately 5000 adults each in Homa Bay and Migori counties in Kenya. Adult residents were tested for HIV through mobile multidisease community health campaigns (CHCs) supplemented by home-based testing (HBT) for campaign nonattendees.^13^

### Study Procedures

On arrival to CHCs, community members received group-based education on PrEP. After HIV testing at CHCs or at home, we assessed HIV acquisition risk among all HIV-negative persons using an empirical risk score, developed by applying machine-learning methods to 2 years of HIV testing data from SEARCH.^14^ The risk score incorporated age, sex, marital status, polygamy, education, circumcision, occupation, and alcohol use, and was calculated at point-of-contact using real-time data entry into tablet computers to generate a dichotomous output (yes/no at risk) that was incorporated into HIV post-test counseling. Participants not assessed “at risk” based empirical data could also self-assess their risk during post-test counseling, as counselors engaged individuals in discussions of their potential risks for HIV (eg, knowledge of partner HIV status, concurrent partnerships, condom use, and circumcision). Individuals who expressed interest in starting PrEP were offered same-day start and a choice to receive continued PrEP at clinic, home, or other community location.

### Population and Measures

PrEP uptake was defined as enrollment into the study and receipt of study-provided medication within 2 weeks. Demographics, circumcision, and alcohol use^15^ were assessed at time of HIV testing; household wealth was calculated based on principal component analysis applied to a survey of household assets.^13^ We reviewed the charts of participants who initiated PrEP to identify perceived risks of HIV and reasons for PrEP initiation. This analysis focused on “older” individuals, defined as persons aged 45 years or older who tested HIV-negative in the 6 Kenyan communities.

### Statistical Analysis

We evaluated the proportion of older HIV-negative individuals who were identified for PrEP by either risk score or self-identified risk and the proportion who initiated PrEP within 14 days of offer of PrEP. Logistic regression was used to identify factors associated with PrEP initiation with analyses stratified by sex to explore sex differences.

### Ethical Approval

The study received ethical approvals from the University of California, San Francisco Committee on Human Research, and the Ethical Review Committee of the Kenya Medical Research Institute.

## RESULTS

A total of 36,573 adults (≥15 years) were enumerated during household census in the 6 study communities; 22% (8038) were aged 45 years and older. Of the 8038 adults aged 45 years and older, 82.3% (6615) received HIV testing, either at a CHC (82.4%, 5453/6615) or subsequent HBT (17.6%, 1162/6615). A total of 5472 older individuals tested HIV-negative and were included in this analysis. Of these, 64% (3407) were women, 62% had attained primary level of education (3,415), and 70% (3845) were married, with 21% of married persons in polygamous relationships.

Among the 5472 HIV-negative older individuals, 401 (7.3%) were at elevated HIV risk and received enhanced counseling on PrEP (265 by risk score, 136 by risk self-identification); 113/265 (43%) identified by the risk score also self-identified as being at elevated risk of HIV acquisition. Of these 401, 143 (36%) initiated PrEP within 2 weeks of testing: 50/265 (19%) identified by risk score and 93/136 (68%) with self-identified risk.

In analyses adjusting for age, occupation, polygamy, residence stability, wealth status, and whether a participant was a head of household, men were more likely to initiate PrEP than women [41% vs. 30%; adjusted odds ratio (aOR) 2.89; 95% confidence interval (CI): 1.33 to 6.29], while those employed in occupations associated with high risk of HIV acquisition such as fishing, transport or entertainment industry was less likely to initiate compared with other occupations including teaching, shop keeping, farming, carpentry, clergy, and tailoring (23% vs. 58%; aOR 0.17; 95% CI: 0.08 to 0.36).

In analyses stratified by gender, men who were heads of households were more likely to initiate PrEP (aOR 2.16; 95% CI: 1.00 to 4.65) than other household members, while among women, being head of a household was not associated with PrEP initiation (aOR 0.72; 95% CI: 0.32 to 1.65) (Table 1). Regardless of gender, those with self-identified risk for HIV were more likely to initiate PrEP compared with those who were identified by empiric risk score (OR 11.4; 95% CI: 5.9 to 21.9 among men; OR 12.7; 95% CI: 6.03 to 26.6 among women) and those HIV tested at home were less likely to initiate PrEP as compared to those who tested at a health campaign (OR 0.05; 95% CI: 0.01 to 0.21 among men; OR 0.02; 95% CI: 0.004 to 0.13 among women).

**TABLE 1.**
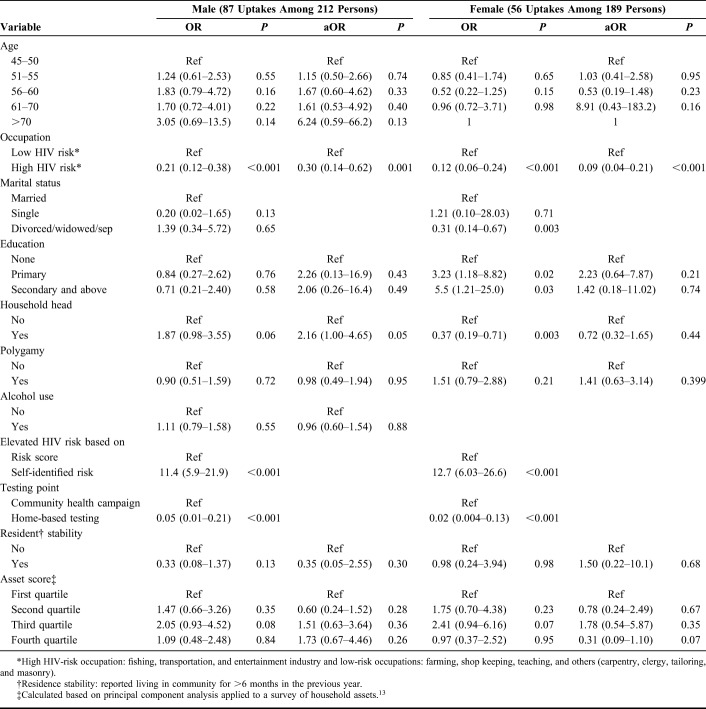
Factors Associated With PrEP Uptake Within 2 Weeks Among 401 HIV-Negative Men and Women Aged 45 Years and Older Assessed at Elevated HIV Risk in Six Rural Kenyan Communities

Based on participant interview abstracted from PrEP enrolment visit chart notes, common reasons to initiate PrEP among the older adults included reporting multiple sexual partners of unknown HIV serostatus (48%), being in a known discordant relationship (22%) or a polygamous marriage (20%), and being an inherited widow or being a wife inheritor (3%).

## DISCUSSION

The findings of our study suggest that there is interest in and willingness to take PrEP among a substantial minority of older individuals at elevated risk of HIV acquisition. More than one-third of older adults identified for PrEP initiated within 2 weeks of HIV testing, a proportion that compares favorably with other studies in younger, targeted populations in Kenya.^16–18^ Given that a substantial proportion of new infections occur among older adults,^8,19^ ensuring access to PrEP beyond currently targeted groups to include older individuals could provide valuable benefit by reducing new infections in this age group and potentially reducing intergenerational and intragenerational HIV transmission. Recent Kenya ARV guidelines in 2018 have changed to recommending PrEP be offered to individuals perceiving themselves at high risk of HIV acquisition,^20^ and this change broadens access to the general population, including older adults. As PrEP is offered to older individuals, tailored sensitization and mobilization strategies and targeted interventions should be developed to foster uptake in this group.

A significant gender difference in uptake of PrEP among this age group was observed. Male participants had higher PrEP uptake compared with female participants, and there were also differences in uptake between male and female heads of household, with male household heads having a higher uptake. This finding highlights the importance of PrEP and other preventive interventions in reducing HIV transmission occurring through intergenerational sex involving younger women. Low PrEP uptake and adherence among women has been observed in other studies that reported underutilization of PrEP as a prevention strategy.^21^ Women continue to bear the brunt of HIV because of vulnerabilities linked to sociocultural, economic, and political inequalities^22^ that are further driven by gender norms related to masculinity vs. femininity, barriers to access to services such as spousal consent, lack of education, and violence against women. These forces may be operating to influence the low uptake of PrEP among our female participants. It is important to improve PrEP uptake among women by addressing barriers to women accessing services with the understanding that women are at a disproportionate risk of acquiring HIV.^23^

Very few individuals who tested at home rather than at CHCs initiated PrEP. We postulate that individual and group education on PrEP that happened at CHC and not at home contributed to a higher uptake at the CHC. In addition, this finding may reflect lower health-seeking behavior in the group tested through HBT as compared to those tested at CHC, although this requires further investigation. We also found that persons in occupations associated with higher risk of HIV acquisition were less likely than those in farming and other professions to initiate PrEP. Previous literature has suggested high levels of HIV stigma in fisherfolk in the Lake Victoria region,^24,25^ which may have impacted PrEP uptake in our study. Individuals identified by risk score were also less likely to uptake PrEP, which may reflect lack of self-perceived risk in this population. Older adults who initiated PrEP in our study reported similar risk behaviors as described among both older and younger adults in previous studies, such as having multiple sexual partners, being in known discordant relationships, and having sexual relations with partners of unknown HIV status.^26,27^ Our findings on the risk behaviors of older adults highlight the importance of making deliberate effort to focus preventive interventions that include this age group.

Our study had some limitations. Although we collected sexual behavior data from PrEP initiators, we did not collect sexual behavior data from all community members, nor did we ask about transactional sex or same-sex partners. Dates of birth were also self reported and may have had some inaccuracies.

In conclusion, one-third of older individuals at elevated risk of HIV expressed interest and willingly engaged in PrEP use as a preventive strategy against HIV infection. Increasing access through targeted interventions for persons aged 45 years and older is warranted, including effort to reduce gender-specific barriers to accessing prevention services, including PrEP. Closing prevention gaps including PrEP among older persons will be an important step toward achieving a comprehensive approach to ending the HIV epidemic.
